# Did the Coronavirus Disease 2019 (COVID-19) Pandemic Cause a Delay in the Diagnosis of Breast Cancer Patients?

**DOI:** 10.7759/cureus.66501

**Published:** 2024-08-09

**Authors:** Bilal Arslan, Erkan Guler, Ahmet Dag, Halil Afsin Tasdelen, Recep Okan Üstün

**Affiliations:** 1 General Surgery, Faculty of Medicine, Mersin University, Mersin, TUR; 2 General Surgery, Trabzon Kanuni Training and Research Hospital, Trabzon, TUR; 3 Plastic and Reconstructive Surgery, Mersin City Hospital, Mersin, TUR

**Keywords:** survival, surgery, neoadjuvant therapy, covid-19 pandemic, breast cancer

## Abstract

Introduction

The coronavirus disease 2019 (COVID-19) outbreak, first reported in Wuhan, China, in December 2019, quickly hit the world in just one month, causing a global public health emergency. We aimed to investigate whether the COVID-19 pandemic caused a delay in the hospital admissions of breast cancer patients and diagnosis of breast cancer, thus increasing the tumor size and the stage of the disease.

Materials and methods

Included in the study were patients who underwent breast cancer surgery between 01/03/2019 and 01/03/2020 (pre-COVID-19, first period) and between 01/03/2020 and 01/03/2021 (post-COVID-19, second period). Three hundred and seventy patients with enough details were included, and details were analyzed retrospectively. Tumor characteristics of pre-COVID-19 breast cancer patients were compared with the tumor characteristics of post-COVID-19 breast cancer patients. Demographics, preoperative diagnosis, tumor properties, surgical procedure (breast-conserving surgery, modified radical mastectomy, simple mastectomy, skin-sparing mastectomy), tumor size, total lymph node number, metastatic lymph node number, locally advanced disease, metastatic disease, and neoadjuvant therapy were evaluated.

Results

The mean tumor size increased significantly in the post-COVID-19 primary surgery group (p=0.005). There is no significant relationship between the pre-COVID-19 and post-COVID-19 period and pT in the neoadjuvant received group (p>0.05). The presence of pT2+pT3+pT4 was statistically significantly higher in the post-COVID-19 primary surgery group (p=0.001). The mean value of metastatic lymph nodes dissected between pre-COVID-19 and post-COVID-19 primary surgery groups increased significantly (p=0.010). Pericapsular extension was higher in the post-primary surgery group (p=0.002).

Conclusion

During the COVID-19 outbreak, breast cancer patients have difficulty accessing healthcare services and hesitate to apply to hospitals to fear contracting the COVID-19 disease. This situation has led to delays in diagnosing breast cancer patients, increased tumor size and pT grade, increased number of metastatic lymph nodes, pericapsular extension, and the resulting disease often appearing in advanced sizes and stages.

## Introduction

The coronavirus disease 2019 (COVID-19) outbreak, first reported in Wuhan, China, in December 2019, quickly hit the world in just one month, causing a global public health emergency [1]. In response to the COVID-19 pandemic, healthcare providers and institutions have recommended delaying the treatment of some cancer patients, especially during peak infection incidence, to reduce patient exposure to the virus and prioritize healthcare resources [2]. However, this situation has caused malfunctions and delays in cancer patients' access to health services, thus delaying cancer patients' treatment. Delays in surgery, chemotherapy, and radiotherapy treatments of cancer patients may worsen the stage of the disease and increase the possibility of metastasis. It has been reported that this situation negatively affects the morbidity and mortality of the disease [3].

Breast cancer is the most common malignancy affecting women worldwide and is the second most common cause of cancer-related death in women. One in every eight women is at risk of developing breast cancer for a lifetime [4]. To detect breast cancer at an early stage, screening methods with proven efficiency are used, which are considered to reduce cancer mortality. Breast imaging studies can be applied as elective screening or diagnostic procedures in suspicious cases. Therefore, screening programs for breast cancer should be implemented for the population [5].

Due to the changing conditions after the COVID-19 pandemic, health institutions started to serve under pandemic conditions [6]. The patients' fear of getting the virus and the change in the priority of health services caused a decline in breast cancer screening program referrals to surgical and genetic counseling units [7]. This is likely to cause a significant decrease in the incidence of early-stage breast cancer detected by screening. There is an increased risk of nodal and distant metastasis for tumors in the 1-5 cm size range (pT1 and pT2). However, evidence shows that the risk of tumor metastasis is related to the biological characteristics of breast cancer rather than tumor size [8]. This study aimed to investigate whether the COVID-19 pandemic causes a delay in the hospital admissions of breast cancer patients and diagnosis of breast cancer, thus increasing the tumor size and the stage of the disease and causing an increase in morbidity and mortality.

## Materials and methods

This study was approved by the Board of Ethics of Mersin University (approval number: 2021/349) on 28/04/2021. Included were patients who underwent breast cancer surgery between 01/03/2019 and 01/03/2020 (pre-COVID-19, first period) and between 01/03/2020 and 01/03/2021 (post-COVID-19, second period). Four hundred and four patients between the ages of 18 and 90 were identified to have breast cancer surgery. Twelve patients with a lack of medical records were excluded. Fifteen patients with distant organ metastasis in the pre-COVID-19 period and 14 patients with distant metastasis in the post-COVID-19 period were sent to medical or radiation oncology clinics, excluded, and evaluated separately. Apart from these distant metastases, three patients in the pre-COVID-19 period and four in the post-COVID-19 period with only bone metastasis gave a complete response and were added to the neoadjuvant therapy group. Three hundred and seventy patients were included in the study, and details were analyzed retrospectively.

To perform clinical staging, tru-cut biopsy for palpable lesions and stereotactic biopsy, wire marking, or roll marking for non-palpable lesions were performed in all patients. The study did not include patients who underwent surgical biopsies in an external center. Mammography (MG) and ultrasonography (USG) were performed in early-stage tumors; bone scintigraphy, cranial MRI, thorax CT, and abdominal USG were performed in symptomatic patients; PET-CT was performed in locally advanced disease; and breast MRI was performed in patients with early-stage tumors suspected of multicentric disease or candidates for breast-conserving surgery.

We evaluated our patients treated for breast cancer one year before the first COVID-19 case was detected in Mersin University, compared with those treated for breast cancer during the past year with the pandemic. Demographics, preoperative diagnosis, tumor properties, tumor size, total lymph node number, metastatic lymph node number, locally advanced disease, metastatic disease, neoadjuvant therapy, and surgical procedure (breast-conserving surgery, modified radical mastectomy) were evaluated.

We aimed to reveal whether there is a delay in diagnosing and initiating treatment in breast cancer patients due to the COVID-19 pandemic. Whether there was an increase in tumor size, number of dissected total axillary lymph nodes, number of dissected metastatic axillary lymph nodes, metastatic disease, and stages of the disease were compared between subgroups due to the delay. All the patients had MG, USG, or MRI in the preoperative period. The same team carried out all the surgical operations. Patient confidentiality is regarded as extremely important at all stages.

Statistical analysis

Descriptive statistics are represented as frequency and percentage. Continuous parameters are defined by average and standard deviation. The Shapiro-Wilk test evaluated the normality control of continuous variables. Since the variables did not conform to a normal distribution, the Mann-Whitney U test was used to compare independent groups. The chi-squared test was used to analyze categorical variables. The IBM SPSS Statistics for Windows, Version 21.0 (Released 2012; IBM Corp., Armonk, New York, United States), was used to analyze the data.

## Results

The mean age of patients was 53.92±12.33. Three hundred and sixty-nine patients were female, and one patient was male. The mean tumor size value was 2.31±1.89. The mean number of dissected total axillary lymph nodes was 10.66±8.86. The mean number of dissected metastatic axillary lymph nodes was 2.46±4.76.

A total of 88 patients received neoadjuvant treatment, 41 in the pre-COVID-19 period and 47 in the post-COVID-19 period.

In the pre-COVID-19 period, 150 patients underwent primary surgical treatment; in the post-COVID-19 period, 132 patients underwent primary surgical treatment, and 282 patients underwent primary surgical treatment.

In the pre-COVID-19 period, two patients had lung metastasis, two patients had liver metastasis, four patients had bone metastasis, two patients had lung+liver metastasis, two patients had liver+bone metastasis, one patient had bone+brain metastasis, and one patient had lung+liver+bone metastasis (total 15 patients, 8%).

Two patients had lung metastasis in the post-COVID-19 period, two patients had liver metastasis, five patients had bone metastasis, one patient had lung+liver metastasis, four patients had liver+bone metastasis, and one patient had lung+liver+bone metastasis (total 14 patients, 7%). When the metastatic disease was compared between groups, there was no significant difference (p>0.05).

Right breast-conserving surgery was performed in 34 patients. Right modified radical mastectomy was performed in 64 patients. Right breast simple mastectomy was performed in 72 patients. Skin-sparing mastectomy and implant procedure were performed in eight of 72 patients. Left breast-conserving surgery was performed in 31 patients. Left-modified radical mastectomy was performed in 81 patients. Left breast simple mastectomy was performed in 88 patients. Skin-sparing mastectomy and implant procedure were performed in nine of 88 patients. In case of any surgical necessity or medical indication, we performed sentinel lymph node biopsy (SLNB) and intraoperative frozen section to detect metastatic disease. If any metastatic disease was detected, we performed axillary lymph node dissection.

In the pre-COVID-19 period, breast-sparing mastectomy was performed in 32 (17.4%) patients. Modified radical mastectomy was performed in 69 (37.7%) patients. Simple mastectomy was performed in 82 (44.8%) patients. In the post-COVID-19 period, breast-sparing mastectomy was performed in 33 (17.6%) patients. Modified radical mastectomy was performed in 76 (40.6%) patients. Simple mastectomy was performed in 78 (41.7%) patients. No significant difference was found among surgical procedures by periods (p>0.05). Demographic and descriptive features of patients are shown in Table 1 and Table 2.

**Table 1:** Demographic features of patients

	Mean	Median	Min-max
Age (year)	53.92±12.33	54 (44-64)	28-93
Tumor size (cm)	2.31±1.89	2.1 (1-3)	0-13
Total lymph nodes (n)	10.66±8.86	10 (2-18)	0-34
Metastatic lymph nodes (n)	2.46±4.76	0 (0-3)	0-28

**Table 2:** Descriptive features of patients

Diagnosis	Pathological subtype (WHO classification, 2019)	n	%
	Invasive ductal	270	73
	Invasive lobular	30	8.1
	Invasive medullary	3	0.8
	Invasive mucinous	6	1.6
	Invasive neuroendocrine	1	0.3
	Invasive papillary	11	3
	Lobular carcinoma in situ	2	5
	Ductal carcinoma in situ	14	3.8
	Metaplastic carcinoma	9	2.4
	Metastatic osteosarcoma	1	0.3
	Mixed carcinoma	22	6
	Paget disease	1	0.3
Breast side	Right	172	46.5
	Left	198	53.5
Surgical procedure	Right breast-conserving surgery	34	9.2
	Right modified radical mastectomy	64	35.7
	Right simple mastectomy	72	1.1
	Left breast-conserving surgery	31	8.4
	Left modified radical mastectomy	81	44.3
	Left simple mastectomy	88	1.4
T stage	pT in situ	17	4.6
	pT1	118	31.9
	pT2	165	44.6
	pT3	25	6.8
	pT4	9	2.4
	pTx (unknown)	36	9.7
Grade	Unknown	54	14.6
	1	112	30.3
	2	146	39.5
	3	58	15.7
Lymphovascular invasion	Unknown	36	9.7
	Yes	128	34.6
	No	206	55.7
Perineural invasion	Unknown	36	9.7
	Yes	71	19.2
	No	263	71.1
Pericapsular invasion	Unknown	36	9.7
	Yes	55	14.9
	No	279	75.4
	Total	370	100

In the neoadjuvant therapy group, the mean tumor size values have changed to decline in the post-COVID-19 group, but not statistically significant (p=0.883). The mean tumor size values in the post-COVID-19 period in the primary surgery group increased significantly (p=0.005).

There was no significant relationship between the pre-COVID-19 and post-COVID-19 period and pT in the neoadjuvant therapy group (p=0.961). There is a significant relationship between the post-COVID-19 period and pT in the patient group who underwent primary surgery (p=0.022). According to this, while pT1 rates were high before COVID-19, it was observed that pT2 rates were higher after COVID-19 (p<0.05). The presence of pT2+pT3+pT4 was significantly higher in patients who underwent primary surgery post-COVID-19 (p=0.001) (Table 3).

**Table 3:** Pathological T stage evaluation of patients treated with neoadjuvant therapy and primary surgery applied p: chi-squared test

Primary therapy type	T stage	Pre-COVID-19		Post-COVID-19		Total		p
		n	%	n	%	n	%	
Neoadjuvant therapy	pT1	11	26.8	12	25.5	23	26.1	0.961
	pT2	10	24.4	11	23.4	21	23.9	
	pT3	3	7.3	5	10.6	8	9.1	
	pTx (unknown)	17	41.5	19	40.4	36	40.9	
Primary surgery	pT in situ	11	7.3	6	4.5	17	6	0.022
	pT1	62	41.3	33	25	95	33.7	
	pT2	67	44.7	77	58.3	144	51.1	
	pT3	7	4.7	10	7.6	17	6	
	pT4	3	2	6	4.5	9	3.2	

There was no statistically significant difference between the pre-COVID-19 and post-COVID-19 neoadjuvant therapy group according to the number of dissected metastatic lymph nodes (p=0.594). When the number of dissected total axillary lymph nodes was evaluated, a significant decrease was observed in the post-COVID-19 neoadjuvant therapy group (p<0.001).

In the primary surgery group, a significant increase was not observed in the number of dissected total axillary lymph nodes between the periods (p=0.350) (Table 4). The mean number of dissected metastatic axillary lymph nodes increased significantly between the periods (p=0.010) (Table 4).

**Table 4:** Size and axillary lymph node evaluation of patients treated with neoadjuvant therapy and primary surgery applied Values in bold mean p<0.05 (statistically significant) p: Mann-Whitney U test; SD: standard deviation; IQR: interquartile range

		Pre-COVID-19			Post-COVID-19			
		Mean±SD	Median (IQR)	Min-max	Mean±SD	Median (IQR)	Min-max	p
Neoadjuvant therapy	Size (cm)	1.48±2.29	0.2 (0-2.1)	0-10	1.40±1.91	0.3 (0-2.5)	0-7	0.883
	Total lymph nodes (n)	16.34±7.88	18 (12.5-21.5)	0-31	9.55±8.54	8 (1-18)	0-28	<0.001
	Metastatic lymph nodes (n)	4.37±6.67	1 (0-7)	0-28	3.11±5.04	1 (0-4)	0-18	0.594
Primary surgery	Size (cm)	2.32±1.57	2 (1.3-3)	0.1-10	2.89±1.86	2.5 (1.8-3.5)	0-13	0.005
	Total lymph nodes (n)	10.48±9.26	7.5 (2-18.25)	0-34	9.5±8.18	7.5 (2-18)	0-26	0.350
	Metastatic lymph nodes (n)	1.46±3.66	0 (0-1)	0-23	2.77±4.86	0 (0-3)	0-20	0.010

There is no significant relationship between periods and grade, lymphovascular invasion, or perineural invasion in the neoadjuvant therapy group or primary surgery group (p>0.05).

There is no significant relationship between the periods and pericapsular extension in the neoadjuvant therapy group (p>0.05). There is a substantial relationship between the periods and pericapsular extension in the primary surgery group (p=0.002). According to this, pericapsular extension presence rates were higher after the COVID-19 outbreak (Table 5).

**Table 5:** Pathological grade of patients treated with neoadjuvant therapy and primary surgery applied p: chi-squared test

Primary therapy type	Grade	Pre-COVID-19		Post-COVID-19		Total		
		n	%	n	%	n	%	p
Neoadjuvant therapy	Unknown	17	41.5	21	44.7	38	43.2	0.559
	1	5	12.2	10	21.3	15	17	
	2	12	29.3	11	23.4	23	26.1	
	3	7	17.1	5	10.6	12	13.6	
Primary surgery	Unknown	12	8	4	3	16	5.7	0.164
	1	54	36	43	32.6	97	34.4	
	2	64	42.7	59	44.7	123	43.6	
	3	20	13.3	26	19.7	46	16.3	

## Discussion

Many healthcare centers worldwide are allocating resources from elective and semi-elective surgical patients to severe COVID-19 patients [9]. To control COVID-19, resource reallocation eventually led to the creation of hospitals partially or dedicated to COVID-19 patients (COVID-19 hospital) [10]. Despite having a separate route and applying infection control (IC) measures during this period, patients could need more time to see a doctor or perform diagnostic procedures due to COVID-19 infection risk, reducing the number of admitted patients in the hospital [11-13].

Without reliable data concerning the end of the pandemic today, the idea of ​​putting everything off until the end of the pandemic is not suitable for cancer patients' treatments. Therefore, several guidelines have been published to reduce the harmful impact of the COVID-19 outbreak [14-16]. Triage of emergency clinical cases, alert, and rapid surgical treatment can increase the number of patients treated and reduce the risk of cross-infection during hospitalization and the period of COVID-19 [17-20].

Breast cancer is a significant public health problem in Turkey as well as all over the world. It is crucial to recognize and diagnose the disease early. Prevention, screening, diagnosis, and treatment of breast cancer include various types of interconnected care, including but not limited to diagnostic breast screening, surgical consultation for radiographic findings, genetic testing and management of gene test results, and treatment [5].

Our study showed a limited increase in the number of patients diagnosed with breast cancer who received neoadjuvant therapy after the COVID-19 pandemic when compared (41 vs. 47). There is a partial decrease in patients who underwent primary surgical treatment (150 vs. 132). No significant difference was found among surgical procedures by periods (p>0.05). We had 369 female and one male breast cancer patients.

Increased tumor size in breast cancer patients may increase the degree of pT, progress the stage of the disease, and cause the tumor to gain an aggressive character. The increased tumor size at the time of diagnosis is associated with increased axillary lymph node involvement and breast cancer-related mortality. A widely accepted theory is that as cancer grows, cells within the tumor spread to regional lymph nodes and other distant sites to gain the ability to develop and survive [21,22]. This view is based on interpreting the well-established relationship between primary tumor size and the prevalence of metastasis. The risk of developing metastases is assumed to increase linearly with tumor size because the more significant the cancer at diagnosis, the more cells are available to metastasize [23].

Some studies have reported contrary opinions, as well. In a retrospective cohort study conducted by Sopik and Narod, the primary tumor size, lymph node involvement, and distant metastasis of 819,647 patients diagnosed with primary invasive breast cancer were examined. Only for tiny tumors (less than 10 mm) and massive tumors (greater than 60-90 mm) was there little correlation between tumor size and the risk of metastasis [24].

We think that avoidance of breast examination may have developed in our country due to the COVID-19 outbreak [25]. A trend in this direction has also been reported in patients from different parts of the world. Nyante et al. reported that, in their study population, the frequency of breast screening and diagnostic examinations was significantly lower than expected after the burst of the COVID-19 pandemic, given the fears that delayed screenings could contribute to delayed cancer diagnoses.

To clarify this situation in our clinic, we wanted to evaluate the pathology results of patients we operated on for breast cancer in the pre-COVID-19 and post-COVID-19 periods. When the patients' pathology results were evaluated numerically regarding tumor size post-COVID-19 period, the mean size value decreased in the neoadjuvant therapy received in the patient group. However, the statistics are not significant (p=0.883). For patients who underwent primary surgery, the mean value of tumor size of breast cancer patients increased in the post-COVID-19 period (p=0.005). While short-term delays in performing an imaging examination or breast biopsy have minimal effects, long-term delays can reduce the number of cancer patients detected in the early stages and potentially increase the number of cancer deaths in the upcoming years [26]. The uncertainty of when the pandemic process will end may present this situation as a more severe problem in the future.

Institutions across the United States have reported reductions in screening examinations among asymptomatic women or diagnostic procedures such as breast biopsies among women with abnormalities [27]. Multiple factors are contributing to the decline in examination applications. One reason may be that patients have stayed at home because of public announcements that caused appointments to be canceled or delayed. To eliminate the possible destructive effects of this situation that may arise over time, effective measures should be taken so as not to hinder the management of the epidemic in our country and all over the world.

In our study, when the tumor sizes were classified according to their pT (TNM system) status, while pT1 rates were high before COVID-19, it was observed that pT2 rates were higher after COVID-19 (p<0.05). However, the presence of pT2+pT3+pT4 (showed the progress of the breast cancer disease) was significantly higher in patients who underwent post-COVID-19 primary surgery (p=0.001). Accordingly, in the post-COVID-19 period, due to the tumor size of breast cancer patients, the pT status has increased considerably compared to the pre-COVID-19 period. In the first months of the pandemic, breast patients refrained from applying to health institutions due to the incomplete understanding of the disease and the fear of getting sick and dying all over the world and in our country. We think that uncertainties about the course and treatment of the disease and the sad images reflected in the worldwide media caused such a situation in breast cancer patients. MG-based population screening and risk-adjusted breast screening programs for asymptomatic subjects (BRCA carriers) have been suspended by international recommendations. However, referrals from general practitioners to hospitals due to suspicious lesions decreased. This situation caused delays in diagnosing breast cancer patients, being evaluated with a multidisciplinary approach, and receiving surgery, neoadjuvant therapy, or radiotherapy.

Axillary lymph node metastasis is the most important predictor of overall recurrence and survival in patients with breast cancer [28]. While the five-year survival rate in patients with disease localized to the breast is 98.8%, this figure drops to 85.8% in patients with regional lymph node metastasis. Therefore, an exact evaluation of the axillary extension is essential in breast cancer's staging and treatment process [29].

There was a statistically significant difference in the mean values of the post-COVID-19 neoadjuvant therapy group according to the number of dissected total axillary lymph nodes (p<0.001). A decline was detected when the number of dissected metastatic lymph nodes was compared. However, no statistically significant decrease was observed in the mean values of the post-COVID-19 neoadjuvant therapy group (p=0.594). This suggests that though the number of patients increased in the post-COVID-19 period, patients gave a better response to neoadjuvant therapy. Moreover, it would be hard to mention a treatment overdue in neoadjuvant therapy received patients. While short-term delays have minimal effects, long-term delays can still deteriorate the disease process. The uncertainty of when the pandemic process will end may present this situation as a more severe problem in the future; currently, it is crucial to handle this situation.

There was no statistically significant difference between the pre-COVID-19 and post-COVID-19 groups, according to the number of total axillary lymph nodes dissected (p=0.350). The mean value of the metastatic axillary lymph nodes increased significantly in the post-COVID-19 period compared to the pre-COVID-19 period (p=0.010). We think this results from patients with breast complaints avoiding examination due to COVID-19. The fear of getting the disease caused a delay in admission in breast cancer patients, leading to increased tumor size and axillary spread of the disease. The stage of the disease has advanced, and patients who can be diagnosed at an early stage have come across as locally advanced. Apart from tumor biology, more aggressive treatment methods were applied to the patient group, which we could cure with less invasive treatment or breast-conserving surgery when detected in smaller sizes.

Pericapsular invasion is often defined as an invasion of at least the lymph node capsule or invasive cancer that passes from the nodal capsule to the perinodal tissue [30].

In this study, there was no statistically significant difference between the groups who received neoadjuvant therapy and underwent primary surgery regarding grade, lymphovascular invasion, and perineural invasion involvement rates between pre-COVID-19 and post-COVID-19 periods (p>0.05). There was no significant relationship regarding pericapsular extension in neoadjuvant therapy received pre-COVİD-19 and post-COVID-19 periods. However, the primary surgery group had a significant relationship between pre-COVID-19 and post-COVID-19 periods and pericapsular extension (p=0.002). According to this, pericapsular extension rates were higher after COVID-19 (Table 5). Pericapsular extension, which is an indicator of the aggressive character of the tumor, increased due to the delay in the referral of patients during the pandemic process.

This study has some limitations. First, it has a single-center, retrospective, non-randomized design with a relatively small sample size. Second, although it revealed that there were delays in accessing treatment for breast cancer patients during the COVID-19 outbreak, it did not provide any conclusions about what precautions should be taken in similar outbreaks. Such an inference could be useful in future outbreaks. One of the study's shortcomings is that the evaluation was not made according to luminal subtypes. For example, it would have been more elegant to compare more aggressive subtypes, such as triple negative, with each other. However, it should be remembered that more patients will be needed for such an evaluation to be meaningful.

It may take many years for the COVID-19 outbreak effects to disappear and for hospitals to return to their regular working order. As health professionals, we should develop methods and take measures to reduce the harmful effects of breast cancer on society and detect breast cancer at earlier stages. During the pandemic, it is recommended that the most consistent regimens and therapeutic sequences be selected to reduce the risk of COVID-19 infection in patients and healthcare workers without compromising the prognosis of patients. In addition, a specialized triage for COVID-19 should be undertaken in all cancer centers before entry; here, patients and family members should be questioned for symptoms, past contact, and fever. In case of suspected COVID-19 infection, patients and accompanying persons should not contact other patients and healthcare professionals.

First, the clinical diagnosis of a breast mass or lump (by self-examination) or any other sign with a high suspicion of malignancy should be evaluated immediately. Similarly, the patients should be evaluated when there is clinical evidence of recurrent locoregional disease. Pathological evaluation is considered a high priority for abnormal mammograms or symptoms in the breast or symptomatic metastatic recurrence. In a pandemic, discussing the risks/benefits of treatments with the patient and obtaining a medical record that clearly states current therapeutic decisions and alternatives is crucial.

## Conclusions

The COVID-19 outbreak devastates patients' and healthcare providers' lives. In this process, cancer patients, in general, and breast cancer patients, in particular, have difficulties accessing healthcare services and hesitate to apply to hospitals for fear of contracting the COVID-19 disease. This situation has led to delays in the diagnosis of breast cancer patients. The COVID-19 pandemic caused a delay in the hospital admissions of breast cancer patients and diagnosis of breast cancer, thus increasing the tumor sizes and the stages of the disease and causing an increase in morbidity. It may take many years for the COVID-19 outbreak's effect on people's lives to disappear and for hospitals to return to their regular working order. As health professionals, we should develop methods and take measures to reduce the harmful effects of breast cancer (a public health problem) on society and detect breast cancer at earlier stages.
